# A case series of myocardial infarction in SARS‐CoV‐2‐infected patients: Same complication, different outcomes

**DOI:** 10.1002/ccr3.5304

**Published:** 2022-01-26

**Authors:** Azin Alizadehasl, Samira Eslami, Kimia Vakili, Shirin Habibi Khorasani, Mehrdad Haghazali, Ehsan khalilipur

**Affiliations:** ^1^ Cardiology Head of Cardio‐Oncology Department and Research Center Rajaie Cardiovascular Medical & Research Center Iran University of Medical Science Tehran Iran; ^2^ Echocardiologist Department of Adult Echocardiography Rajaie Cardiovascular Medical and Research Center Tehran Iran; ^3^ Student Research Committee Faculty of Medicine Shahid Beheshti University of Medical Sciences Tehran Iran; ^4^ Echocardiologist Department of Adult Echocardiography Rajaie Cardiovascular Medical and Research Center Tehran Iran; ^5^ Gastroenterologist Rajaie Cardiovascular Medical and Research Center Iran University of Medical Sciences Tehran Iran; ^6^ Interventional Cardiologist Cardiovascular Intervention Research Center Rajaie Cardiovascular Medical and Research Center Tehran Iran; ^7^ Present address: Shahid Rajaei Cardiovascular Training Research and Treatment Center St. Tehran Iran

**Keywords:** COVID‐19, myocardial infarction (MI), SARS‐CoV‐2, thrombosis

## Abstract

Thrombosis is frequently observed in COVID‐19, especially in critically ill patients. Management of such life‐threatening conditions is of high importance in the context of the current pandemic. Herein, we provide a case series of myocardial infarction in the clinical evolution of COVID‐19, emphasizing its importance and implications on the cardiovascular system.

## Abstract 2

Due to proinflammatory state in COVID‐19, myocardial infarction may occur. In addition to its fatal entity, the challenges at the way of acute management of myocardial infarction at the time of COVID‐19 pandemic have worsened the outcome of patients.

## 1 INTRODUCTION

The newly emerged coronavirus disease 2019 (COVID‐19) has started a drastic pandemic since its first emergence in December 2019 in Wuhan, China.^1^ A huge body of evidence has shown that in addition to the conventional respiratory (dyspnea, cough, etc.) and constitutional (fever, malaise, etc.) symptoms, cardiovascular manifestations including thrombotic events are not uncommon and may be associated with serious complications.^2^ These thrombotic events can present in different forms such as venous thromboembolism (VTE),^3^ pulmonary embolism (PE),^4^ cerebrovascular accident (CVA),^5^ acute myocardial infarction (MI),^6^ renal artery thrombosis,^7^ and mesenteric ischemia.^8^ Although the occurrence of thrombosis as a result of COVID‐19 infection has been reported, the exact cause of this co‐occurrence is still under investigation. Pathological studies have suggested different mechanisms such as diffuse microthrombi^9^ and complement‐mediated microvascular injury.^10^

ACE2 receptors, majorly found in endothelial cells, provide the main pathway for SARS‐CoV‐2 viral entry and lead to endothelitis.^11^ Additionally, systemic inflammatory response that increases the level of procoagulants (correlated with acute phase reactants) is one of the main contributor to thrombogenesis.^12^ To be more detailed, the hypercytokinemia caused by inflammatory response can cause endothelial damage that exposes tissue factor and leads to activation of the coagulation cascade. This activation generates a hypercoagulability state, where thromboembolic events and ultimately disseminated intravascular coagulation (DIC) occurs.^13^ However, despite the lack of adequate evidence, many studies have reported the correlation between acute myocardial infarction and SARS‐CoV‐2 infection.^14^, ^15^

In addition to pathophysiology, challenges in the way of appropriate management of these patients are of high importance. It is well established that the prognosis of patients with acute myocardial infarction is strongly associated with the speed of diagnosis and early treatment.^16^ There are several factors leading to late admission to the hospital, including patient‐related, healthcare system–related, and logistical factors.^17^, ^18^ Following the emergence of COVID‐19 pandemic, studies have shown the reduced ED visits for life‐threatening conditions^19^; particularly, they suggest that the number of patients presenting with acute MI has decreased.^20^ This might be because of the fear of acquiring the disease in healthcare facilities and governmental social distancing measures.^20^ Unfortunately, despite decreased myocardial infarction referrals to the hospitals, the incidence of cardiac arrest has surged in hard‐hit areas, showing that how fatal this healthcare avoidance could be. In this paper, we have reported a series of MI cases with concomitant COVID‐19 infection, admitted from April to July 2020 which fully describes the challenges in the management of patients with life‐threatening emergencies at the COVID‐19 era.

## 2 CASE 1

**FIGURE 1:**
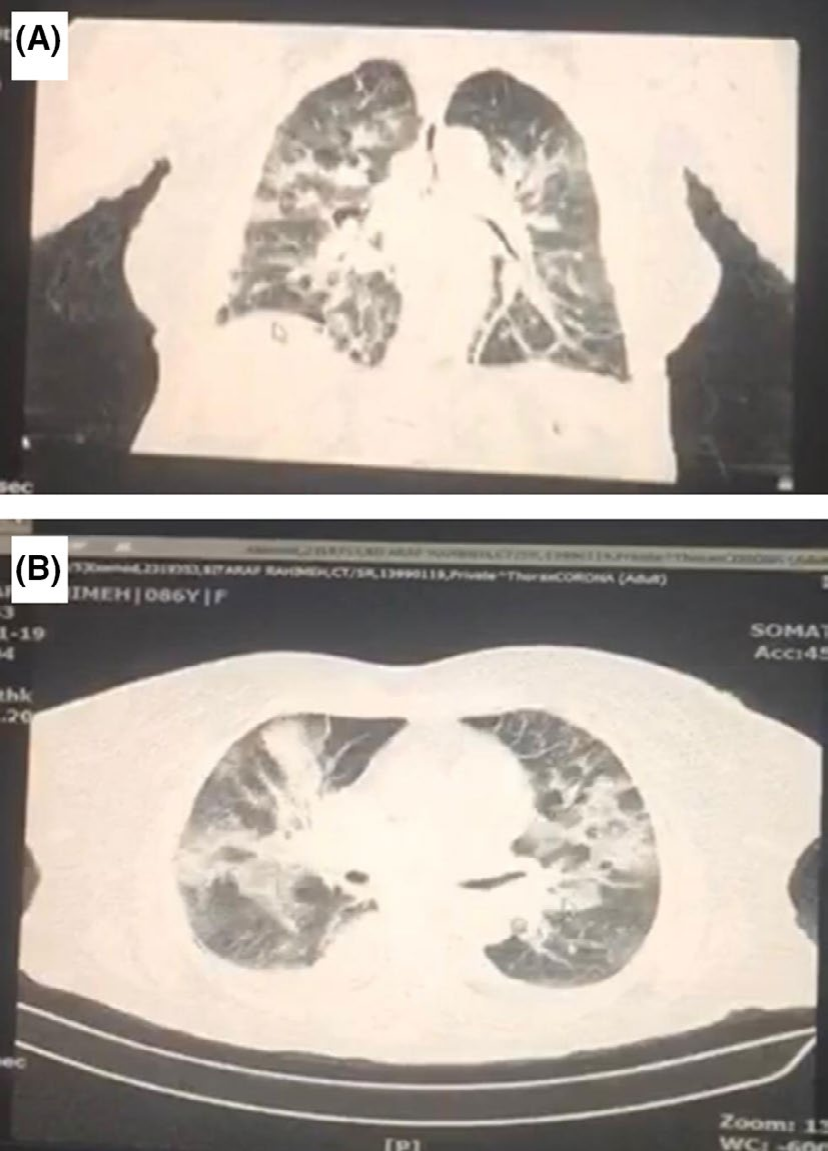
(A&B) Chest CT scan revealed bilateral consolidation

An 80‐year‐old lady with a previous history of diabetes and hypertension, presented with compressive chest pain for 10 h prior to admission. Although she denied any respiratory or flu‐like symptoms at the time, due to COVID‐19‐related protocols, diagnostic COVID‐19 work‐ups were conducted for the patient. As a result, chest X‐ray and CT scan depicted bilateral parenchymal consolidation with approximately 50% involvement of the lung parenchyma with SARS‐CoV‐2 (Figure 1). On admission, vital signs were stable and physical examination revealed no significant abnormality. The electrocardiogram (ECG) showed ST‐segment elevation in inferior leads and ST‐segment depression in V2‐V4. Transthoracic echocardiography (TTE) showed concordant regional wall motion abnormality and reduction in left ventricular systolic function. Due to concomitant COVID‐19 infection and the 10‐h delay in patient's referral, she was not scheduled for primary PCI. The patient was admitted in COVID‐19 intensive respiratory care unit and pharmacological treatment started. The acute cardiac ischemia medications included tablet ASA 80 mg daily, tablet Plavix 75 mg daily, tablet atorvastatin 80 mg daily, tablet captopril 25 mg daily, and amp enoxaparin 60 mg twice daily. Following several hours, amp furosemide (IV infusion) was started due to occurrence of bilateral fine rales. The COVID‐19 medications included hydroxychloroquine and Kaletra (lopinavir/ritonavir). Laboratory tests were as follows: serum creatinine level = 1.1 mg/dl, blood sugar = 285 mg/dl, WBC = 10.600/mm^3^, CRP = 7.8 mg/L, and elevated troponin I (5.45 ng/ml).

In the first 24 h, the chest pain resolved and the hemodynamic status remained stable. Thereafter, due to O2 saturation level drop to 88%, non‐invasive ventilation (NIV) with BiPAP was started, and subsequently, the O2 saturation was raised to 95%.

On the second day, the hemodynamics were stable and NIV was not further indicated; so that she was switched to O2 therapy with face mask. On the third day, the rise in PCO2 resulted in re‐prescription of NIV and addition of bronchodilators. At the same time, ECG showed evolving inferior MI with normal QT interval. Despite the NIV, the rise in PCO2 persisted and the patient got intubated on the fourth day due to decreased O2 saturation level and loss of consciousness. Following the start of mechanical ventilation, the patient developed bradycardia, asystole, and cardiac arrest within few hours. Unfortunately, the CPR was unsuccessful and the patient died.

## 3 CASE 2

**FIGURE 2:**
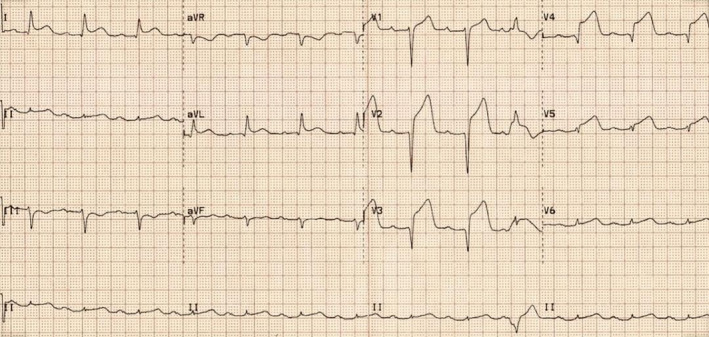
Electrocardiogram showed ST‐elevation MI and first‐degree AV block

**FIGURE 3:**
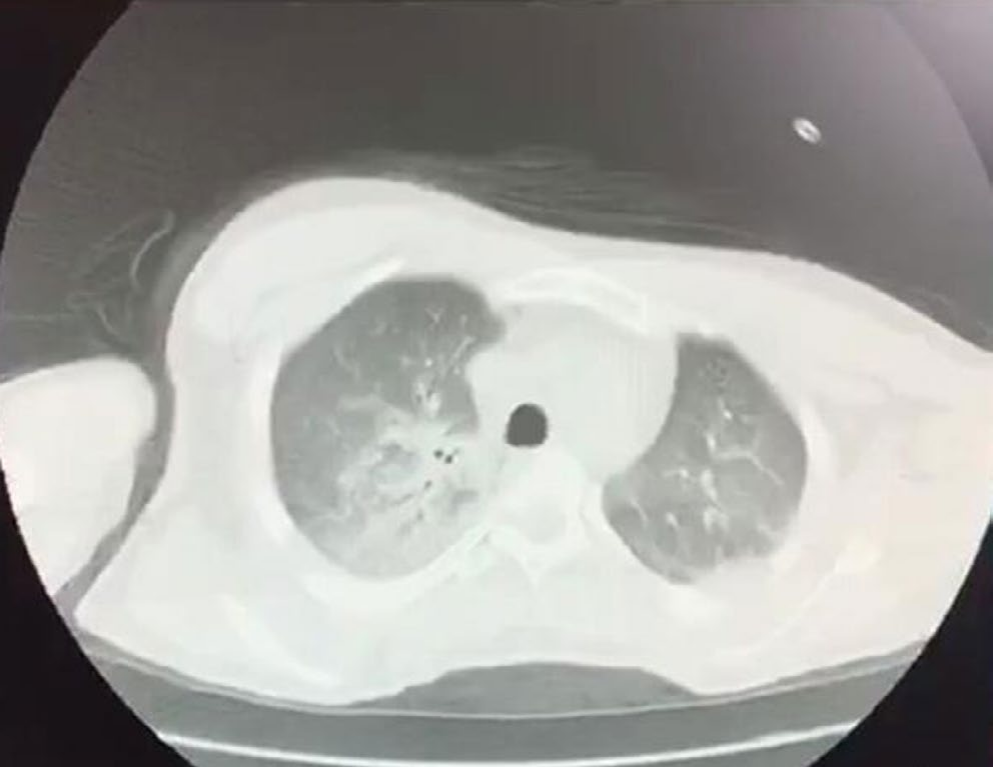
Chest CT scan showing bilateral pulmonary infiltration suggestive of COVID‐19 pneumonia

**FIGURE 4:**
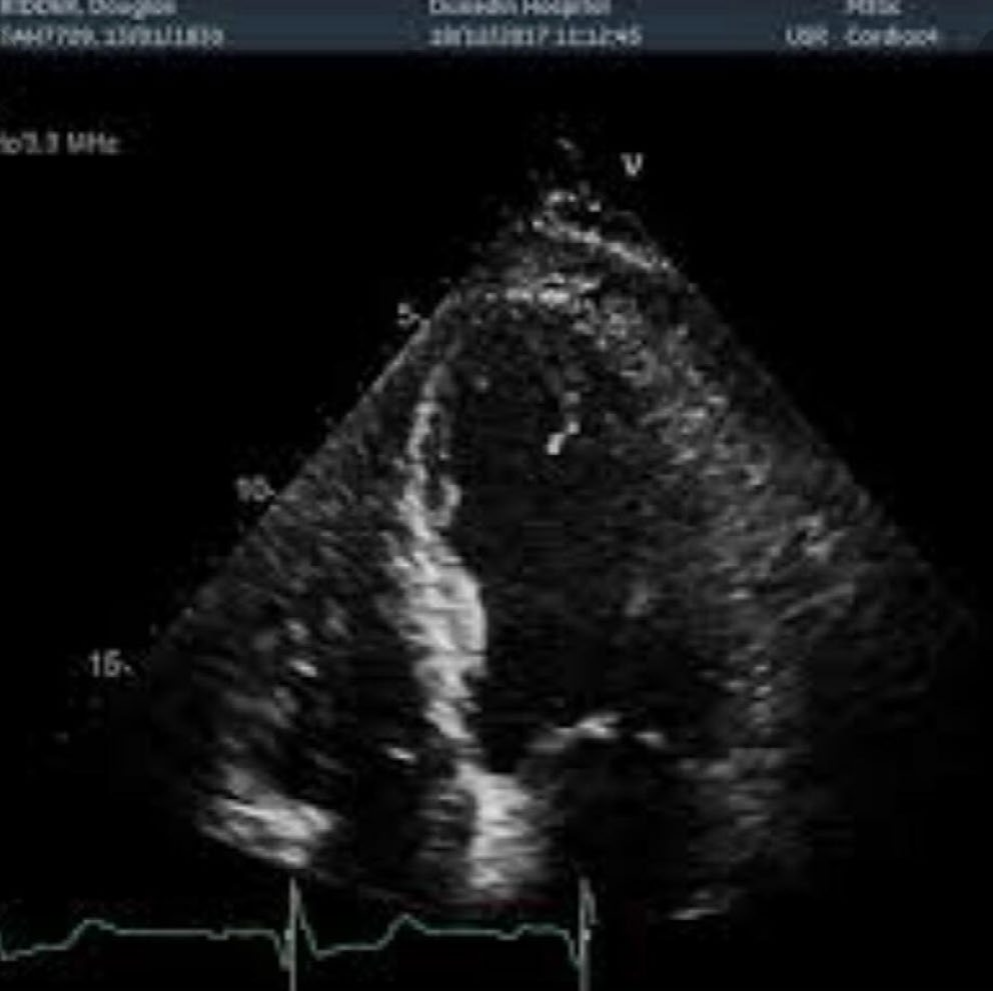
Transthoracic echocardiography revealed anterior wall and apical segments akinesia consistent with anterior MI

**FIGURE 5:**
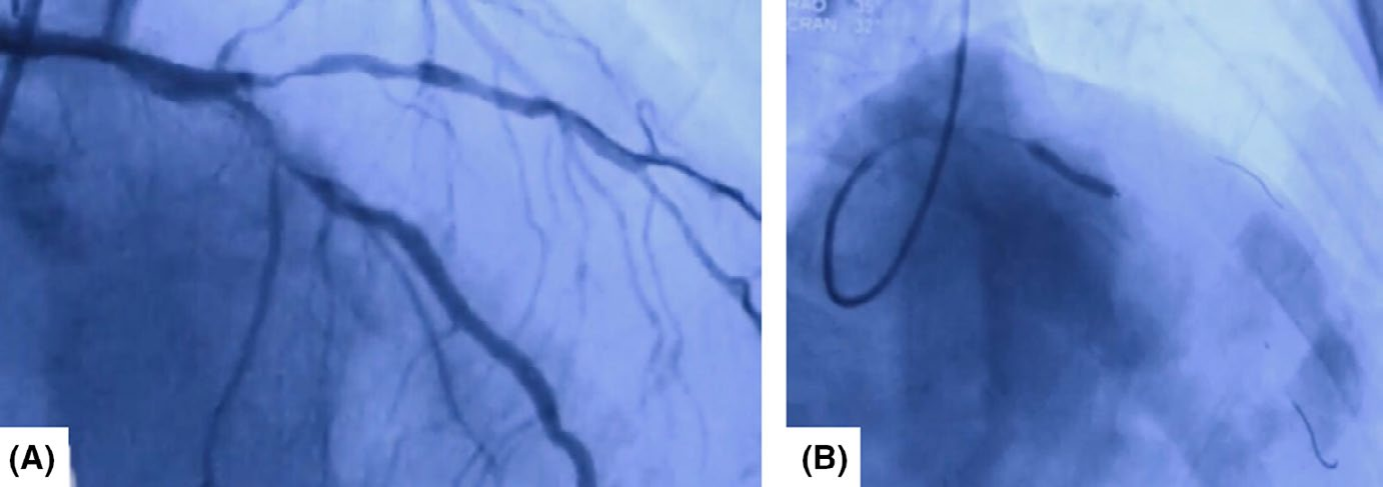
Coronary angiography revealed significant stenosis in the proximal part of LAD (A), for which successful PCI was performed (B)

A 45‐year‐old man with previous history of hyperlipidemia was referred to emergency department due to dyspnea, chest pain, fever, chills, and dry cough. He mentioned flu‐like symptoms in the past couple of days. Vital signs were as follows: blood pressure = 110/90 mmHg, heart rate = 113 bpm and oxygen saturation in room air = 90%, and temperature = 38.5°C orally. 12‐lead ECG showed extensive anterior ST‐segment elevation MI and first‐degree AV block, suggestive of transmural myocardial infarction (Figure 2). Chest CT scan revealed bilateral subpleural patchy consolidations associated with pleural effusion (Figure 3). Transthoracic echocardiography (TTE) revealed left ventricular systolic dysfunction, with an estimated LV ejection fraction of 30%. There was no evidence of significant valvular involvement (Figure 4). Laboratory tests were as follows: troponin I level = 17.9 ng/L (normal value <50 ng/L), elevated CRP and ESR. Nasopharyngeal swab (PCR) test confirmed SARS‐Cov‐2 infection. Medical therapy was started for the patient and included dual antiplatelet therapy (tablet ASA 80 mg daily + tablet Plavix 75 mg daily) alongside with tablet Metoprolol 50 mg and tablet atorvastatin 80 mg daily. Regarding the findings and active chest pain at the time of arrival, the patient was scheduled for primary PCI. Coronary angiography demonstrated thrombotic occlusion of proximal portion of LAD and first diagonal. The patient underwent successful PCI on LAD and was transferred to CCU (Figure 5, A&B). Clinical improvement was satisfactory, and the patient discharged 7 days after admission.

## 4 CASE 3

**FIGURE 6:**
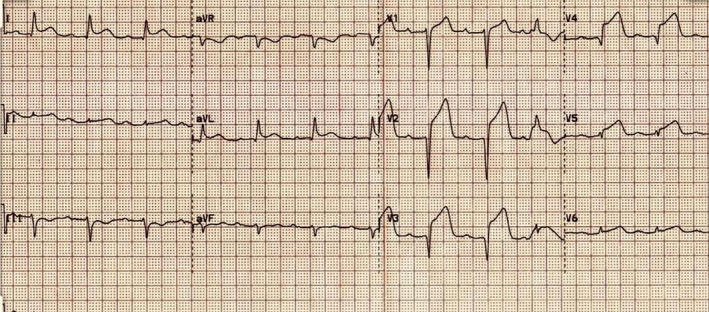
Electrocardiogram showing anterolateral MI

**FIGURE 7:**
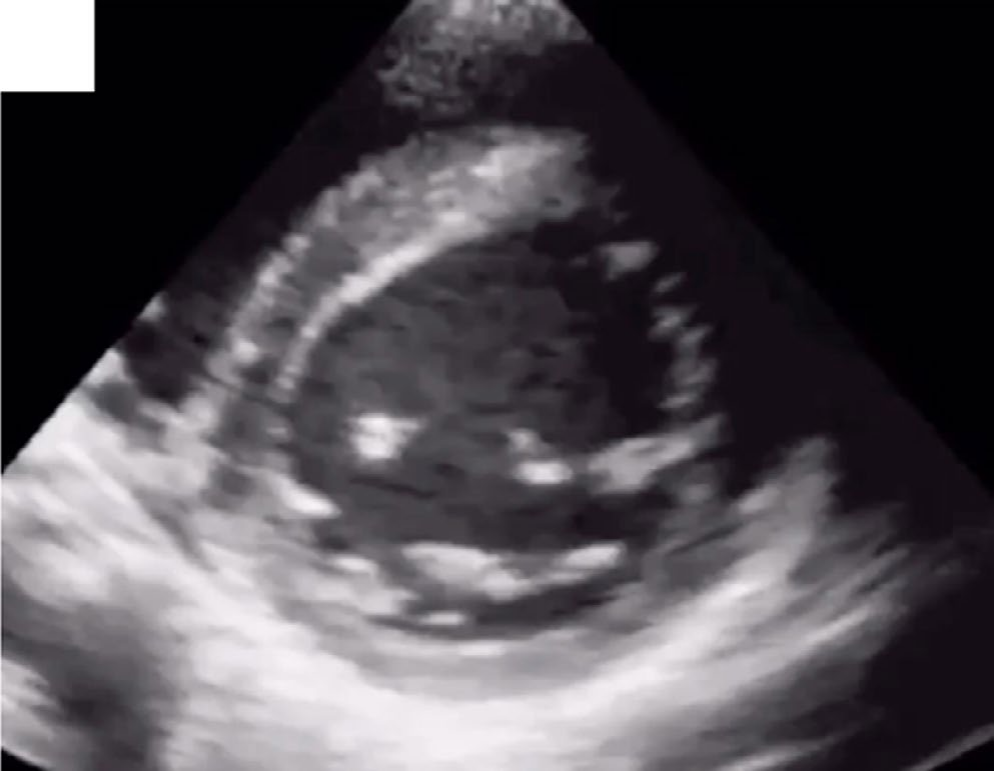
Echocardiography showed hypokinesia in anterior circulation with severe reduction in left ventricular systolic function

**FIGURE 8:**
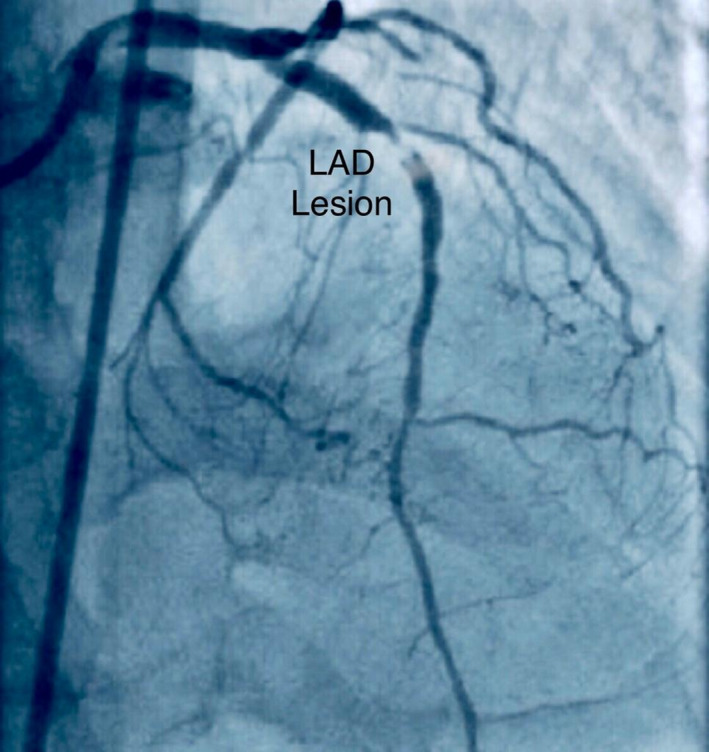
Coronary angiography showed significant lesion in proximal part of LAD

A 39‐year‐old man with history of hypertension who mentioned dyspnea, severe chest pain, and sweating in the past 4 days that became intolerable, so that he was made to visit emergency room. He stated active COVID‐19 infection in his first‐degree relative who lived with him. At time of admission, the vital signs were as follows: blood pressure = 110/80 mmHg, PR = 110 bpm, and O2 saturation in room air = 92%. Electrocardiogram revealed anterolateral MI (Figure 6), accompanied with concordant wall motion abnormality in LAD circulation territory in transthoracic echocardiography (Figure 7). The laboratory tests showed elevated troponin I level and positive COVID‐19 PCR. Emergency coronary angiography depicted occlusive LAD thrombus, for which primary PCI was performed and resulted in reconstitution of left coronary artery flow (Figure 8). The prescribed medications included aspirin, clopidogrel, atorvastatin, azithromycin, and hydroxychloroquine. Kaletra was not recommended for the patient regarding acute ischemic phase. In few hours, oxygen saturation dropped to 78% and the patient developed respiratory acidosis (pH = 7.47). The emergent chest X‐ray showed diffuse bilateral opacities in favor of COVID‐19 pneumonia. The subsequent respiratory failure resulted in endotracheal intubation and invasive mechanical ventilation. On the third day of admission, the patient developed with QT prolongation (about 500 ms); therefore, azithromycin was discontinued. However, hydroxychloroquine administration continued with close ECG monitoring. With optimal ICU care, the patient could make through extubation and was discharged about a month following admission.

## 5 DISCUSSION

COVID‐19 is a disease with a wide spectrum of symptoms and clinical manifestations, ranging from no symptoms to acute respiratory distress syndrome (ARDS) requiring mechanical ventilation.^21^ An early report from Wuhan, China, declared that the rate of myocardial injury (based on elevation of biomarkers) in COVID‐19 patients is about 12%.^22^ Another study, reported a risk of 8% for acute cardiac injury in COVID‐19, with a 13‐fold higher incidence in critically ill patients.^23^ Thrombotic events are well‐known complications of COVID‐19 infection.^23^ Like our patients, there are several reports in literature, which have discussed acute myocardial infarction arising in the setting of COVID‐19.^2^, ^23^, ^24^

The severity of clinical manifestations of COVID‐19 may vary at each stage^21^: (1) mild phase (resolving in ~80% of the cases) within first 7 days in which most of the symptoms are due to upper respiratory tract infection (ageusia, anosmia, gastrointestinal disturbances and etc.), (2) moderate pneumonia (occurring in ~15% of the cases) from the 10th day in which symptoms worsen and progress to lower respiratory tract (cough, dyspnea, O2 saturation drop, increase in inflammatory indicators i.e., CRP, D‐dimer, ferritin and prothrombotic component and CT findings), (3) severe pneumonia (evolving in ~5% of the cases) in which respiratory condition deteriorates and leads to hypoxemia. The main pathophysiological finding at this stage is cytokine storm and further hypercytokinemia.^13^ This hypercytokinemic state can lead to endothelial damage and consequent activation of coagulation cascade.

In addition to the prominent role of inflammation, other predisposing factors include immobilization, hypoxemia, and rarely disseminated intravascular coagulation (DIC).^15^, ^25^ It has been declared that the risk of thrombotic events in COVID‐19 patients is higher than hospitalized patients with severe pneumonia due to other causes.^25^ Bioinformatics studies have found that during COVID‐19 infection, several viral proteins and glycoproteins can bind to both β‐chain of hemoglobin and porphyrin; resulting in serum hemoglobin drop and hypoxemia. This impaired gas exchange can contribute to extensive lung inflammation.^26^

The important thing that should be kept in mind is that the antiviral medications that are prescribed for COVID‐19 infection may have significant cardiovascular effects. For example, as azithromycin and hydroxychloroquine can both prolong QT intervals resulting in arrhythmogenicity (especially malignant ventricular arrhythmias such as Torsades de pointes).^15^, ^27^ Lopinavir/Ritonavir (Kaletra), which is primarily an anti‐retroviral drug, is under investigation as a potential COVID‐19 medication. This drug may also prolong the QT interval, especially in those with underlying QT abnormalities.^28^ Furthermore, Kaletra can interfere with anticoagulant therapy pharmacodynamics and deteriorate hypercoagulability state in which acute ischemia may occur.^29^, ^30^

In addition to the pathophysiology of the virus and the impact of antiviral medications, the other factor that can dramatically affect the prognosis of ACS patients in the context of COVID‐19 pandemic is the acute management of the disease. Different studies in different countries have been conducted depicting the importance of this acute management. A study conducted by Erol et al. in Turkey^16^ revealed that there is a significant delay in treatment of acute MI during the COVID‐19 pandemic. They have stated that despite on‐time performance of PCI (i.e. door‐to‐balloon time was not prolonged), the significantly increased risk of major adverse cardiac events (MACE) during the pandemic era might be related to patient‐related delays (e.g. fear of acquiring the disease in healthcare facilities). In another paper from India, written by Ramakrishnan et al.,^31^ the same decrease in acute MI‐related hospital admissions has been reported, which is enriched by some interesting hypotheses from the authors. They have proposed two main causes for this dropped rate of admission: (1) decreased access to the hospital because of either unavailability of transportation (which is an issue in low‐middle income countries like India) and fear of COVID‐19 contact in hospitals or (2) a truly decreased ACS incidence due to reduced air pollution and job‐related stresses. However, according to the evidences, the impact of COVID‐19 on acute MI care seems to be the core reason. There are also other studies from other countries indicating the same dropped admission rate,^19^, ^32^, ^33^ which emphasizes on the fact that this issue must be taken seriously as a global concern. We are also facing an increasing number of case reports of late presentations of MI such as ventricular septal defects, myocardial ruptures, and ventricular pseudo‐aneurisms pointing out to unfortunate sequalae of such delays.^34^, ^35^ At last, it must be noted that a special attention must be devoted to vulnerable patients, especially patients with comorbidities requiring anticoagulant treatments. These patients are mostly in need of routine visits to the healthcare facilities, which is associated with higher risk of exposure to the virus. In cases of these patients, a wise switch of therapeutic choice, proper patient education or using telemedicine and remote monitoring measures would be viable options.^36^, ^37^

In conclusion, it can be conceived that due to hypercytokinemia and proinflammatory state in COVID‐19, thrombosis either venous or arterial may occur. Myocardial infarction is a known complication with this pathophysiology and associated with worse outcome and higher mortality rate. Concomitant myocardial ischemia and COVID‐19 infection would deprive the patient from receiving antiviral drugs. It is of great importance to closely monitor the ECG and patients' condition for timely management of these complications. In addition, the acute management of this life‐threatening condition must be taken seriously for which a proper patient education—especially in high‐risk population—can be very helpful.

## CONFLICT OF INTEREST

The authors declare that there is no conflict of interest.

## AUTHOR CONTRIBUTIONS

Azin Alizadehas: Conceptualization, Data Curation, and Writing‐Review and Editing. Samira Eslami: Supervision, Data Curation, and Writing‐Original Draft. Kimia Vakili: Writing‐Original Draft. Shirin Habibi Khorasani, Mehrdad Haghazali, and Ehsan khalilipur: Writing‐Review and Editing.

## CONSENT

Written informed consent was obtained from the patient to publish this report in accordance with the journal's patient consent policy.

## Data Availability

Data sharing not applicable to this article as no datasets were generated or analyzed during the current study.
